# The Successes and Failures of the Initial COVID-19 Pandemic Response in Romania

**DOI:** 10.3389/fpubh.2020.00344

**Published:** 2020-07-10

**Authors:** Stefan Dascalu

**Affiliations:** ^1^Department of Zoology, University of Oxford, Oxford, United Kingdom; ^2^Avian Influenza Group, The Pirbright Institute, Woking, United Kingdom

**Keywords:** Romania, COVID-19, coronavirus, pandemic, epidemic, public health, diaspora, sociocultural

## Abstract

In the context of the COVID-19 pandemic, countries around the world varied in the strength and timeliness of their responses. In Romania, specific challenges were faced with regards to managing the spread and limiting the impact of the disease, ranging from healthcare infrastructure to demographic and sociocultural aspects. As the country has a sizeable diaspora, major difficulties were faced when large numbers of individuals from highly affected areas returned to Romania. However, the fast implementation of control measures successfully averted a surge in the number of COVID-19 cases. This delayed the overburdening of an already challenged healthcare system during the initial phases of the epidemic. Furthermore, early control was facilitated by the exploitation of communication channels that penetrated all layers of society, from ordinary citizens to governmental authorities and high-ranking religious figures. The management of the COVID-19 crisis in Romania illustrates the importance of a fast initial response which takes into account the role played by sociocultural aspects in the context of an epidemic. As the challenges faced by Romania are not unique, these results could inform future public health strategies worldwide.

## Introduction

The severe acute respiratory syndrome coronavirus 2 (SARS-CoV-2), the causative agent of coronavirus disease 2019 (COVID-19), has rapidly spread across the world. On 11th March 2020, the disease was declared a pandemic by the World Health Organization (WHO) (1). In response to this, most countries concentrated their initial efforts on blocking transmission by quarantining confirmed cases and isolating contacts. With the number of infected individuals increasing rapidly, governments began to employ more restrictive procedures concerning the activities and movements of the general population. However, the effectiveness of these responses varied both between and within countries due to several factors such as timeliness, preparedness of healthcare systems, and even sociocultural aspects that influenced the awareness and compliance of the public.

In Romania, the authorities followed WHO recommendations, thereby implementing measures in a similar manner to other countries that were affected by the novel coronavirus (2). However, challenges were faced at various stages of epidemic control, including large numbers of citizens returning from abroad, inadequate healthcare system infrastructure, and sociocultural determinants. In this paper, the successes and failures of the COVID-19 response in Romania are presented in order to inform future strategies in the context of an ongoing pandemic. As important lessons can be learned from the series of events which unfolded in Romania, parallels could be drawn in order to aid the management of future public health crises.

## The Early Stages of the Romanian COVID-19 Epidemic

On 26th February 2020, the first case of COVID-19 was confirmed in Gorj county in south-western Romania (3). The infected individual had been in close contact with an Italian national who visited Romania between 18th and 22th February and was tested positive upon returning to Italy. Indeed, it was the Italian authorities that alerted their Romanian counterparts, thereby facilitating the rapid identification and isolation of contacts, some of whom later tested positive for SARS-CoV-2. Moreover, as the first patient did not exhibit any symptoms of COVID-19, a potential scenario of superspreading was avoided (4).

**Figure 1:**
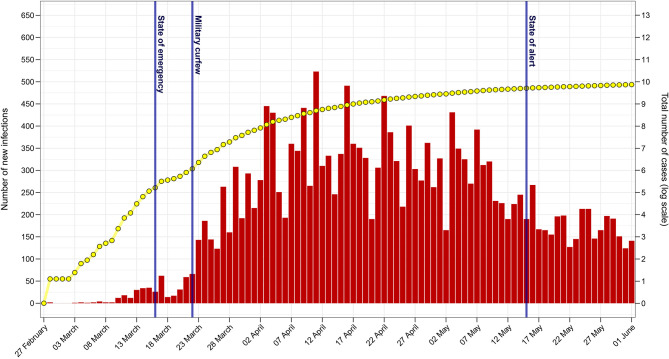
The Romanian COVID-19 epidemic from 27th February to 1st June 2020. The daily number of new confirmed infections is shown in red and the total number of cases (log scale) is displayed in yellow. Notable governmental actions with regards to the epidemic are shown in blue. The figure was created using data from the daily reports released by the Romanian Ministry of Internal Affairs (available in Supplementary Table 1).

Although “patient zero” was identified early on in Romania, other cases soon followed which were not epidemiologically linked to the first infections. By 14th March, Romania exceeded 100 confirmed cases, most of them being Romanian citizens who were returning from highly affected areas (5). Shortly thereafter, local transmission surpassed the importation of cases (6). In response to the increasing number of infections, a state of emergency was declared on 16th March, whereby certain rights such as freedom of movement were limited and non-essential businesses were closed (Figure 1) (7). On 25th March, Romania increased these restrictions by instituting a military curfew (8). The new measures included confining citizens over the age of 65 to their homes and reducing the daily movement of the population to a minimum, such as essential shopping and visiting pharmacies or hospitals. On 26th March, the total number of confirmed cases exceeded 1,000, and there were serious concerns that the healthcare system would be overwhelmed if the infection rate continued to increase. Indeed, the country started facing severe problems in managing the rising number of COVID-19 patients.

## Returning Citizens and the Importation of COVID-19 Cases Into Romania

Romania has one of the largest diasporas in the European Union (EU), with more than three million citizens living abroad (9). Due to the economic uncertainties and panic-stricken atmosphere associated with the COVID-19 pandemic, many expatriates returned to Romania, especially from severely affected countries such as Italy or France (7). As human migratory movement was shown to be a good predictor of the magnitude of an early epidemic, the successive waves of returning citizens posed an enormous challenge to border control and healthcare authorities (10). This problem needed to be addressed rapidly, as the initial stages of spread were crucial in determining the outcomes of epidemic control. Furthermore, the concerns of the public about these issues required careful management, as improper communication had the potential to result in a stigmatization of returning expatriates (11, 12).

In March 2020 alone, more than 250,000 Romanians returned from abroad, and the numbers were projected to increase in the weeks before Easter (13). As an early response, all Romanian nationals who were returning from regions affected by COVID-19 were required to sign a declaration of compliance to a 14 day period of self-isolation (2, 7). However, many citizens did not report truthfully about where they were returning from, with some even taking diverted routes to enter Romania (7). A dramatic example was the case of a retired officer who lied about not having traveled abroad when he was hospitalized (14). By the time he was diagnosed with COVID-19, he had infected at least 30 people, including medical personnel. Furthermore, there were countless examples of people disregarding the official guidelines by participating in large social gatherings (15). This resulted in hundreds of people being quarantined after coming into contact with confirmed patients.

In response to the widespread disregard of official guidelines, the authorities introduced mandatory self-isolation or institutionalized quarantine for those returning from moderately or severely affected countries, respectively (16). An important aspect is that the police escorted the majority of returning citizens to quarantine facilities in their home counties, thereby avoiding an overwhelming of infrastructure in the border regions (17). However, these actions also influenced public opinion, as Romanian expatriates were blamed and vilified for spreading COVID-19 (18). In response to this, messages targeted at both the local population and returning citizens were delivered by government authorities and public personalities, including religious figures (19, 20). These actions not only informed the public about the COVID-19 epidemic, but also limited the stigmatization of returning expatriates. Moreover, by raising awareness, these measures likely reduced the contribution of imported cases to the initial spread of SARS-CoV-2. Indeed, these early interventions were vital in reducing the impact of the challenges that were soon faced throughout the country.

## Responses of the Romanian Healthcare System

In Romania, the majority of medical services are provided through the public healthcare system, which still largely relies on old infrastructure built under the former communist regime (21). In addition, Romania has the lowest health expenditure of all EU countries in both percentage of gross domestic product (GDP) and per capita expenditure (22). These shortcomings already caused issues during previous epidemics and emergency situations, especially due to a lack of necessary equipment, inadequate medical facilities, and insufficient supplies (23, 24). Furthermore, the Romanian healthcare system also faces the problem of understaffing due to the continuous emigration of medical personnel. Consequently, many rural areas have no proximal access to healthcare units, with the closest medical facilities often being located at considerable distances (25). Together, these pre-existing issues exacerbated the challenges posed by the COVID-19 pandemic.

By the end of March 2020, an increasing number of Romanian hospitals were treating COVID-19 cases (26). As infections started to be reported among medical personnel, many wards were closed and patients with other conditions were either transferred within the same hospital or to other medical facilities. However, in many situations, this type of response was not employed early enough. As a consequence, infections started spreading rapidly among healthcare workers and patients, resulting in hospitals and even entire cities being quarantined.

A dramatic example of such a scenario occurred at the county hospital of Suceava, in north-eastern Romania (27). There, improper management coupled with a lack of protocols and inadequate protective equipment resulted in an explosion of COVID-19 cases. In addition, the hospital management attempted to hide the irregularities and not report the initial cases among medical personnel. Sadly, patients with critical conditions were infected and many of them died due to complications associated with COVID-19. Consequently, on 30th March, as more and more cases were being reported, the government decided that Suceava and several neighboring localities were to enter complete lockdown. Additionally, the administration of the county hospital was taken over by the military, which acquired appropriate protective equipment and instituted strict protocols for all medical personnel. The unfortunate series of events that unfolded in Suceava demonstrates the crucial role of the management body of medical institutions during an ongoing public health crisis. Furthermore, it also shows the need for elaborate protocols and emergency provisions of medical supplies in order to avoid potential tragedies.

In light of events such as the one in Suceava, severe concerns arose about supplies of protective equipment and the availability of medical resources. Although healthcare authorities reiterated that sufficient provisions exist and/or that new orders are on the way, the situation in local hospitals did not reflect this (28). Consequently, because of the dangerous conditions in which they were required to work, several doctors and nurses across the country presented their formal resignation (29). Their concerns were not limited to the safety of the healthcare workers themselves, but also included the well-being of patients who were not yet infected with SARS-CoV-2. As some of these resignations came into effect, alarms were raised at a nation-wide scale, prompting the local and national authorities to accelerate the provisioning of necessary resources to handle the epidemic.

At the same time, concerns arose with regards to the effects of widespread resignations on the already understaffed healthcare system. These waves of resignations, which were intended to raise awareness of issues in the healthcare system, created a public outcry (29, 30). Indeed, these actions gave the impression of patients being abandoned in a time of uttermost need. As a first response, government officials debated the withdrawal of rights to practice medicine from the resigning healthcare workers (30). This highlighted the challenges faced by medical personnel in reporting issues within the healthcare system. It further demonstrated that hasty and uncoordinated actions may lead to public unrest, thereby emphasizing the responsibilities held by medical staff outside of their standard professional settings during health crises.

## The Importance of Sociocultural Factors in Epidemic Control

Sociocultural determinants specific to Romania influenced the management and control of the COVID-19 pandemic. Extensive corruption has been plaguing the Romanian healthcare system for years, which has resulted in the population losing trust in its services (21, 24, 31). As a consequence, the prospect of being denied essential care and/or acquiring nosocomial infections upon hospitalization deterred people from seeking medical aid (32).

In order to raise awareness about COVID-19, the Romanian authorities initiated a nationwide information campaign through various media channels, including television and social media (33). This encouraged preventive measures such as social distancing, the wearing of face masks and the use of disinfectants. In addition, as more restrictive measures had to be enforced, the necessity of these actions was clarified by politicians and healthcare officials on a regular basis.

However, the numerous fines issued by the authorities during the initial stages of the epidemic illustrated the reluctance of many citizens to comply with the restrictions to their daily lives (34). Situations of public unrest also became apparent, especially where quarantine centers for suspected COVID-19 patients were opened (35). In some cases, violent outbreaks were only narrowly avoided. These situations were mainly caused by confusing and often poorly delivered information which did not take into account specific sociocultural determinants. Romania has one of the highest levels of poverty, social exclusion, and restricted access to education in the EU, and any public information campaigns would have needed to consider these aspects (36).

Romania also ranks among the most religious countries in the EU, with over 80% of its population identifying as Orthodox Christian (24, 37). While most major religions and other Christian denominations in Europe swiftly announced measures in response to the pandemic, the European Orthodox Churches were among the slowest to respond, partly due to their highly conservative doctrines (38). Indeed, Orthodox rituals and ceremonies had the potential to dramatically increase COVID-19 transmission events, as they often involve large gatherings of people and close contact between believers. However, despite shortcomings and delays, the Romanian Orthodox Church followed the recommended measures and advised all believers and clerics to take appropriate precautions (38, 39). In addition, through the media channels of the Church, governmental advice was reiterated alongside traditional religious guidance, including content directly addressing returning emigrants (20). These communications and further efforts were the result of an active dialogue between experts and high-ranking Church officials. Furthermore, in order to support the collective efforts to reduce the burden of the pandemic, the Church adapted pre-existing structures to offer charitable aid to the vulnerable and even create quarantine centers for COVID-19 patients (39). This demonstrates the need for healthcare authorities to establish efficient channels of communication, which penetrate all layers of society, and subsequently exploit them during public health crises. When combined with conventional control measures, these approaches will simultaneously inform and guide public opinion with regards to healthcare issues.

## Conclusion

The challenges faced during the early stages of the COVID-19 epidemic in Romania have revealed valuable insights into areas which are in need of major improvements. The most apparent issues were found within the healthcare system, where a combination of mismanagement, ignored concerns of medical staff, and inadequate infrastructure drastically weakened the potential of the pandemic response. However, by promptly addressing these key issues, a scenario of uncontrolled spread was avoided. The rapid employment of containment measures coupled with the dissemination of information through all available channels was instrumental in reaching this outcome. Indeed, by early May, the increase in incidence in Romania was comparable to that of moderately affected countries (40). As such, on 15th May, the authorities replaced the national state of emergency with a state of alert, thereby relaxing most of the restrictions (see Figure 1). Despite this largely positive outcome, the COVID-19 pandemic has highlighted areas of the Romanian healthcare system where improvements are required in order for similar scenarios to be successfully managed in the future. Nevertheless, the early stages of this crisis illustrated the importance of both reacting rapidly and considering specific sociocultural aspects in the context of an epidemic. Indeed, such future approaches will make the best use of the available resources and channels of communication during an ongoing health crisis.

## Author Contributions

The author confirms being the sole contributor of this work and has approved it for publication.

## Conflict of Interest

The author declares that the research was conducted in the absence of any commercial or financial relationships that could be construed as a potential conflict of interest.
