# Supplementary material for: The Successes and Failures of the Initial COVID-19 Pandemic Response in Romania
Source: Front Public Health. 2020 Jul 10;8:344. doi: 10.3389/fpubh.2020.00344 (PMC7381272; doi:10.3389/fpubh.2020.00344)
Supplement: Supplementary file 1 [file Table_1.DOCX]

**Supplementary Table 1:** The Romanian COVID-19 Epidemic. Data obtained from the reports released by the Romanian Ministry of Internal Affairs.

| **Date** | **Total cases** | **New cases** | **Total deaths** | **New deaths** |
| --- | --- | --- | --- | --- |
| 27 February 2020 | 1 | 1 | 0 | 0 |
| 28 February 2020 | 3 | 2 | 0 | 0 |
| 29 February 2020 | 3 | 0 | 0 | 0 |
| 01 March 2020 | 3 | 0 | 0 | 0 |
| 02 March 2020 | 3 | 0 | 0 | 0 |
| 03 March 2020 | 4 | 1 | 0 | 0 |
| 04 March 2020 | 6 | 2 | 0 | 0 |
| 05 March 2020 | 7 | 1 | 0 | 0 |
| 06 March 2020 | 9 | 2 | 0 | 0 |
| 07 March 2020 | 13 | 4 | 0 | 0 |
| 08 March 2020 | 15 | 2 | 0 | 0 |
| 09 March 2020 | 17 | 2 | 0 | 0 |
| 10 March 2020 | 29 | 12 | 0 | 0 |
| 11 March 2020 | 47 | 18 | 0 | 0 |
| 12 March 2020 | 59 | 12 | 0 | 0 |
| 13 March 2020 | 89 | 30 | 0 | 0 |
| 14 March 2020 | 123 | 34 | 0 | 0 |
| 15 March 2020 | 158 | 35 | 0 | 0 |
| 16 March 2020 | 184 | 26 | 0 | 0 |
| 17 March 2020 | 246 | 62 | 0 | 0 |
| 18 March 2020 | 260 | 14 | 0 | 0 |
| 19 March 2020 | 277 | 17 | 0 | 0 |
| 20 March 2020 | 308 | 31 | 0 | 0 |
| 21 March 2020 | 367 | 59 | 0 | 0 |
| 22 March 2020 | 433 | 66 | 2 | 0 |
| 23 March 2020 | 576 | 143 | 4 | 2 |
| 24 March 2020 | 762 | 186 | 8 | 4 |
| 25 March 2020 | 906 | 144 | 13 | 5 |
| 26 March 2020 | 1029 | 123 | 17 | 4 |
| 27 March 2020 | 1292 | 263 | 24 | 7 |
| 28 March 2020 | 1452 | 160 | 29 | 5 |
| 29 March 2020 | 1760 | 308 | 38 | 9 |
| 30 March 2020 | 1952 | 192 | 46 | 8 |
| 31 March 2020 | 2245 | 293 | 69 | 23 |
| 01 April 2020 | 2460 | 215 | 85 | 16 |
| 02 April 2020 | 2738 | 278 | 94 | 9 |
| 03 April 2020 | 3183 | 445 | 116 | 22 |
| 04 April 2020 | 3613 | 430 | 141 | 25 |
| 05 April 2020 | 3864 | 251 | 148 | 7 |
| 06 April 2020 | 4057 | 193 | 157 | 9 |
| 07 April 2020 | 4417 | 360 | 182 | 25 |
| 08 April 2020 | 4761 | 344 | 209 | 27 |
| 09 April 2020 | 5202 | 441 | 229 | 20 |
| 10 April 2020 | 5467 | 265 | 257 | 28 |
| 11 April 2020 | 5990 | 523 | 282 | 25 |
| 12 April 2020 | 6300 | 310 | 306 | 24 |
| 13 April 2020 | 6633 | 333 | 318 | 12 |
| 14 April 2020 | 6879 | 246 | 322 | 4 |
| 15 April 2020 | 7216 | 337 | 362 | 40 |
| 16 April 2020 | 7707 | 491 | 387 | 25 |
| 17 April 2020 | 8067 | 360 | 400 | 13 |
| 18 April 2020 | 8418 | 351 | 417 | 17 |
| 19 April 2020 | 8746 | 328 | 434 | 17 |
| 20 April 2020 | 8936 | 190 | 451 | 17 |
| 21 April 2020 | 9242 | 306 | 482 | 31 |
| 22 April 2020 | 9710 | 468 | 508 | 26 |
| 23 April 2020 | 10096 | 386 | 527 | 19 |
| 24 April 2020 | 10417 | 321 | 552 | 25 |
| 25 April 2020 | 10635 | 218 | 575 | 23 |
| 26 April 2020 | 11036 | 401 | 608 | 33 |
| 27 April 2020 | 11339 | 303 | 631 | 23 |
| 28 April 2020 | 11616 | 277 | 650 | 19 |
| 29 April 2020 | 11978 | 362 | 675 | 25 |
| 30 April 2020 | 12240 | 262 | 695 | 20 |
| 01 May 2020 | 12567 | 327 | 726 | 31 |
| 02 May 2020 | 12732 | 165 | 755 | 29 |
| 03 May 2020 | 13163 | 431 | 780 | 25 |
| 04 May 2020 | 13512 | 349 | 803 | 23 |
| 05 May 2020 | 13837 | 325 | 827 | 24 |
| 06 May 2020 | 14107 | 270 | 858 | 31 |
| 07 May 2020 | 14499 | 392 | 876 | 18 |
| 08 May 2020 | 14811 | 312 | 898 | 22 |
| 09 May 2020 | 15131 | 320 | 926 | 28 |
| 10 May 2020 | 15362 | 231 | 952 | 26 |
| 11 May 2020 | 15588 | 226 | 972 | 20 |
| 12 May 2020 | 15778 | 190 | 991 | 19 |
| 13 May 2020 | 16002 | 224 | 1016 | 25 |
| 14 May 2020 | 16247 | 245 | 1046 | 30 |
| 15 May 2020 | 16437 | 190 | 1056 | 10 |
| 16 May 2020 | 16704 | 267 | 1081 | 25 |
| 17 May 2020 | 16871 | 167 | 1097 | 16 |
| 18 May 2020 | 17036 | 165 | 1107 | 10 |
| 19 May 2020 | 17191 | 155 | 1126 | 19 |
| 20 May 2020 | 17387 | 196 | 1141 | 15 |
| 21 May 2020 | 17585 | 198 | 1151 | 10 |
| 22 May 2020 | 17712 | 127 | 1159 | 8 |
| 23 May 2020 | 17857 | 145 | 1170 | 11 |
| 24 May 2020 | 18070 | 213 | 1179 | 9 |
| 25 May 2020 | 18283 | 213 | 1193 | 14 |
| 26 May 2020 | 18429 | 146 | 1210 | 17 |
| 27 May 2020 | 18594 | 165 | 1219 | 9 |
| 28 May 2020 | 18791 | 197 | 1229 | 10 |
| 29 May 2020 | 18982 | 191 | 1240 | 11 |
| 30 May 2020 | 19133 | 151 | 1253 | 13 |
| 31 May 2020 | 19257 | 124 | 1262 | 9 |
| 01 June 2020 | 19398 | 141 | 1270 | 8 |
| 02 June 2020 | 19517 | 119 | 1279 | 9 |
| 03 June 2020 | 19669 | 152 | 1288 | 9 |
| 04 June 2020 | 19907 | 238 | 1299 | 11 |
| 05 June 2020 | 20103 | 196 | 1308 | 9 |
| 06 June 2020 | 20290 | 187 | 1318 | 10 |
